# Supplementary material for: Concentric hollow multi-hexagonal platelets from a small molecule
Source: Nat Commun. 2024 Jul 6;15:5668. doi: 10.1038/s41467-024-49995-3 (PMC11227555; doi:10.1038/s41467-024-49995-3)
Supplement: Supplementary file 1 — Supplementary Information [file 41467_2024_49995_MOESM1_ESM.pdf]

Supplementary Information

Concentric Hollow Multi-Hexagonal Platelets from A Small Molecule

Chenglong Liao,[1,2,#] Yanjun Gong,[1,2,#] Yanxue Che,[3] Hongwei Ji,[1,2] Bing Liu,[2,4,*] Ling Zang,[5,*] Yanke Che,[1,2,*] and Jincai Zhao[1,2]

[1] Key Laboratory of Photochemistry, CAS Research/Education Center for Excellence in Molecular Sciences, Institute of Chemistry, Chinese Academy of Sciences, Beijing 100190, China.

[2] University of Chinese Academy of Sciences, Beijing 100049, China.

[3] HT-NOVA Co., Ltd., Zhuyuan Road, Shunyi District, Beijing 101312, China.

[4] Beijing National Laboratory for Molecular Sciences, State Key Laboratory of Polymer Physics and Chemistry, Institute of Chemistry, Chinese Academy of Sciences, Beijing 100190, China.

[5] Nano Institute of Utah, and Department of Materials Science and Engineering, University of Utah, Salt Lake City, UT 84112, United States.

*Correspondence to: liubing@iccas.ac.cn; lzang@eng.utah.edu; ykche@iccas.ac.cn.

Table of Contents

1. Synthesis of molecules **1** and **2**

2. Supplementary figures 7 to 24

3. Additional supplementary figure

4. Supplementary references

1. Synthesis of molecules 1 and 2

1.1 Synthesis of molecule 2

Molecule **2** was synthesized by following the previously reported method.[1] The detailed synthesis method are as follows:

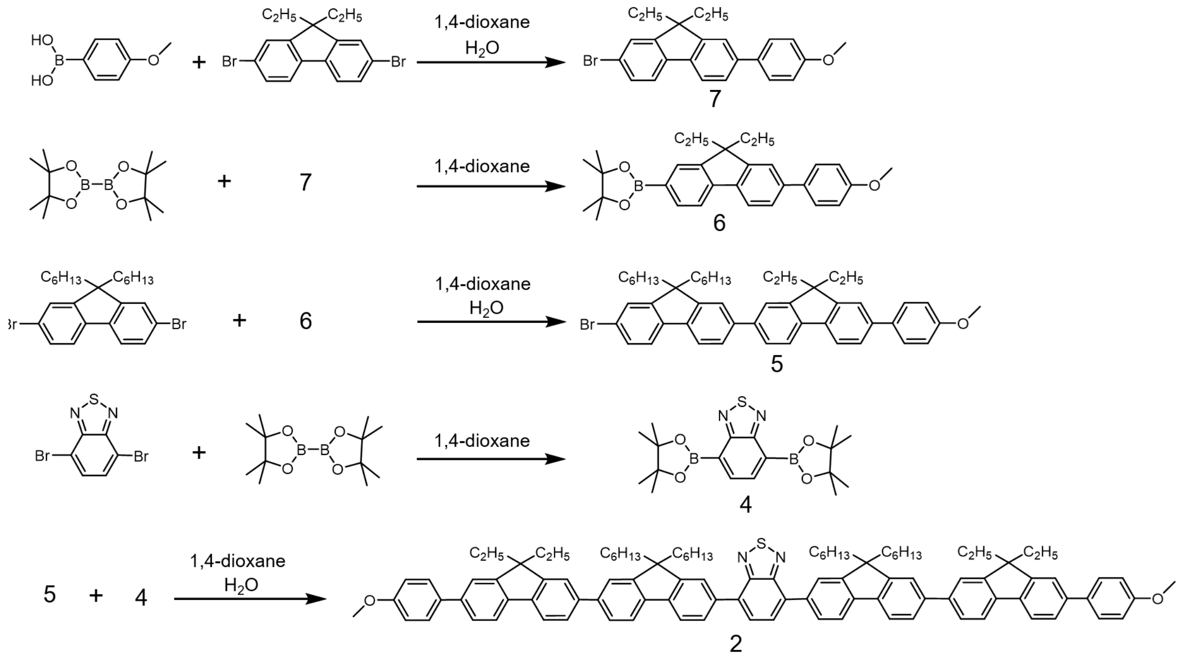

Supplementary Figure. 1 Synthesis route of molecule **2**.

2-bromo-9,9-diethyl-7-(4-methoxyphenyl)-9H-fluorene (7). To a stirred solution of (4-methoxy-phenyl)boronic acid (1 g, 6.57 mmol), 2,7-dibromo-9,9-diethyl-9H-fluorene (3 g, 7.9 mmol), and tetrakis(triphenylphosphine)palladium (380 mg, 0.33 mmol) in deoxygenated 1,4-dioxane (40 mL), an aqueous solution of potassium carbonate (8 mL, 4.0 M) was added. Then the mixture was heated to 80 °C and stirred under argon overnight. The solvent was then evaporated under vacuum. The residue was poured into water (40 mL) and extracted with dichloromethane (3 × 40 mL). The combined organic layer was washed with brine (saturated), dried over Na_2SO_4, and concentrated under vacuum. The resulting residue was purified by column chromatography on silica gel (petroleum: dichloromethane = 20:1 as the eluent) to afford **7** (2.15 g, 5.26 mmol, 80% yield).

2-(9,9-diethyl-7-(4-methoxyphenyl)-9H-fluoren-2-yl)-4,4,5,5-tetramethyl-1,3,2-dioxaborolane (6). A mixture of **7** (2.14 g, 5.26 mmol), 2,7-dibromo-9,9-dihexyl-9H-fluorene (2.68 g, 10.52 mmol), potas-sium acetate (2.58 g, 26.4 mmol), and Pd(dppf)Cl_2 (0.39 g, 0.53 mmol) in deoxygenated 1,4-dioxane (40 mL) was stirred at 85 °C under argon for 8 h. After removal of the solvent under vacuum, the residue was poured into water (40 mL) and extracted with ethyl acetate (3 × 45 mL). The combined organic layer was washed with brine (saturated), dried over Na_2SO_4, and concentrated under vacuum. The residue was

purified by column chromatography on silica gel (petroleum: dichloromethane = 5:1 as the eluent) to afford **6** (1.68 g, 3.69 mmol, 70% yield).

7-bromo-9',9'-diethyl-9,9-dihexyl-7'-(4-methoxyphenyl)-9H,9'H-2,2'-bifluorene (5). To a stirred solution of **6** (1.68 g, 3.69 mmol), 2,7-dibromo-9,9-dihexyl-9H-fluorene (2.18 g, 4.43 mmol), and tetrakis(triphenylphosphine)palladium (213 mg, 0.18 mmol) in deoxygenated 1,4-dioxane (40 mL), an aqueous solution of potassium carbonate (8 mL, 4.0 M) was added. Then the mixture was heated to 80 °C and stirred under argon overnight. The solvent was evaporated under vacuum. The residue was poured into water (40 mL) and extracted with dichloromethane (3 × 40 mL). The combined organic layer was washed with brine (saturated), dried over Na_2SO_4, and concentrated under vacuum. The residue was purified by column chromatography on silica gel (petroleum: dichloromethane = 15:1 as the eluent) to afford **5** (1.9 g, 2.58 mmol, 70% yield).

4,7-bis(4,4,5,5-tetramethyl-1,3,2-dioxaborolan-2-yl)benzo[c][1,2,5]thiadiazole (4). A mixture of 4,7-dibromobenzo[c]-1,2,5-thiadiazole (0.46 g, 1.58 mmol), potassium acetate (0.5 g, 4.75 mmol), bis(pinacolato)diboron (1 g, 3.95 mmol), and Pd(dppf)Cl_2 (0.12 g, 0.16 mmol) in deoxygenated 1,4-dioxane (40 mL) was stirred at 80 °C under argon for 8 h. After removal of the solvent under vacuum, the residue was poured into water (40 mL) and extracted with ethyl acetate (3 × 45 mL). The combined organic layers were washed with brine (saturated), dried over Na_2SO_4, and concentrated under vacuum. The residue was purified by column chromatography on silica gel (petroleum: ethyl acetate = 20:1 as the eluent) to afford **4** (0.48 g, 1.23mmol, 77% yield).

4,7-bis(9',9'-diethyl-9,9-dihexyl-7'-(4-methoxyphenyl)-9H,9'H-[2,2'-bifluoren]-7-yl)benzo[c][1,2,5]thiadiazole (2). To a stirred solution of **5** (1.9 g, 2.58 mmol), **4** (0.48 g, 1.23 mmol), and tetrakis(triphenylphosphine)palladium (142 mg, 0.123 mmol) in deoxygenated 1,4-dioxane (40 mL), an aqueous solution of potassium carbonate (8 mL, 4.0 M) was added. Then the mixture was heated to 80 °C and stirred overnight under argon. The solvent was evaporated under vacuum. The residue was poured into water (40 mL) and extracted with dichloromethane (3 × 40 mL). The combined organic layer was washed with brine (saturated), dried over Na_2SO_4, and concentrated under vacuum. The residue was purified by column chromatography on silica gel (petroleum: dichloromethane = 3:1 as the eluent) to afford **2** (1.15 g, 0.80 mmol, 65% yield).

The resulting target molecule **2** was confirmed by ^{1}H NMR and MALDI-MS as below.

Molecule **2**. ^{1}H NMR (400 MHz, Chloroform-*d*) δ 8.06 (d, J = 7.8 Hz, 2 H), 8.00 (s, 2 H), 7.86 (m, 10 H), 7.61 (m, 16 H), 7.02 (d, J = 8.2 Hz, 4 H), 3.89 (s, 6 H), 2.16 (d, J = 10.1 Hz, 16 H), 1.23 – 1.02 (m, 24 H), 0.87 (d, J = 9.0 Hz, 8 H), 0.78 (t, J = 6.6 Hz, 12 H), 0.46 (t, J = 7.3 Hz, 12 H). MALDI-MS: (m/z) = 1454.26.

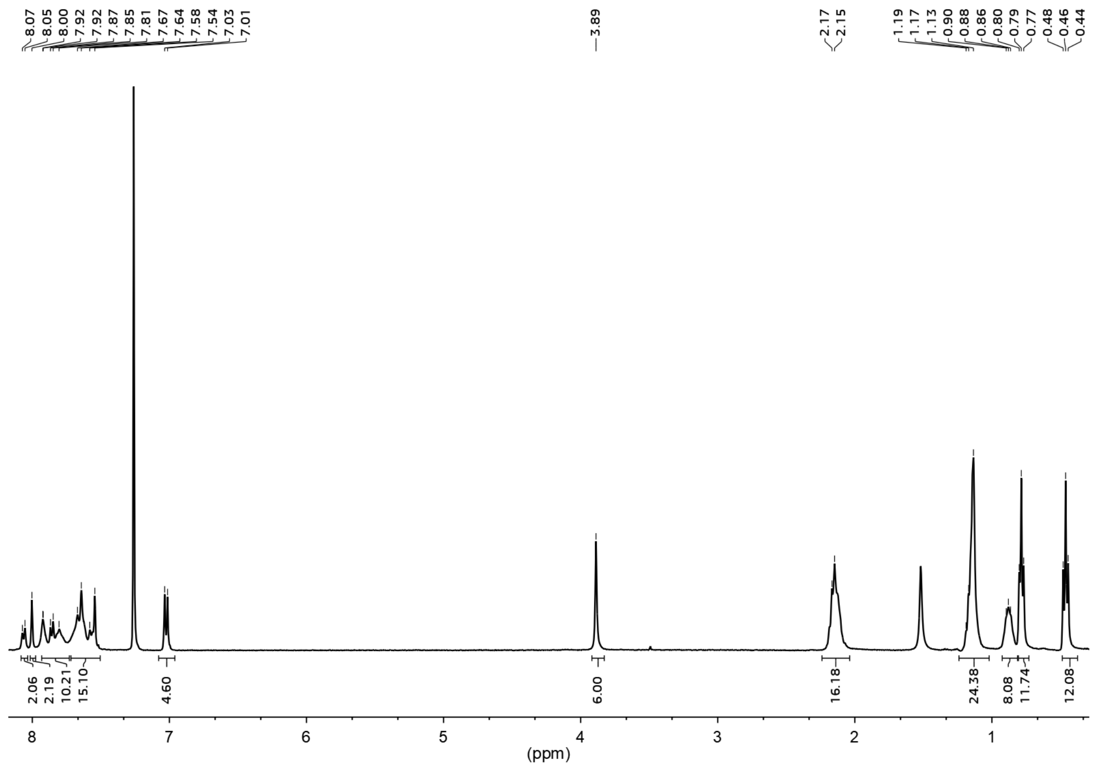

Supplementary Figure 2. ^{1}H NMR of molecule **2**.

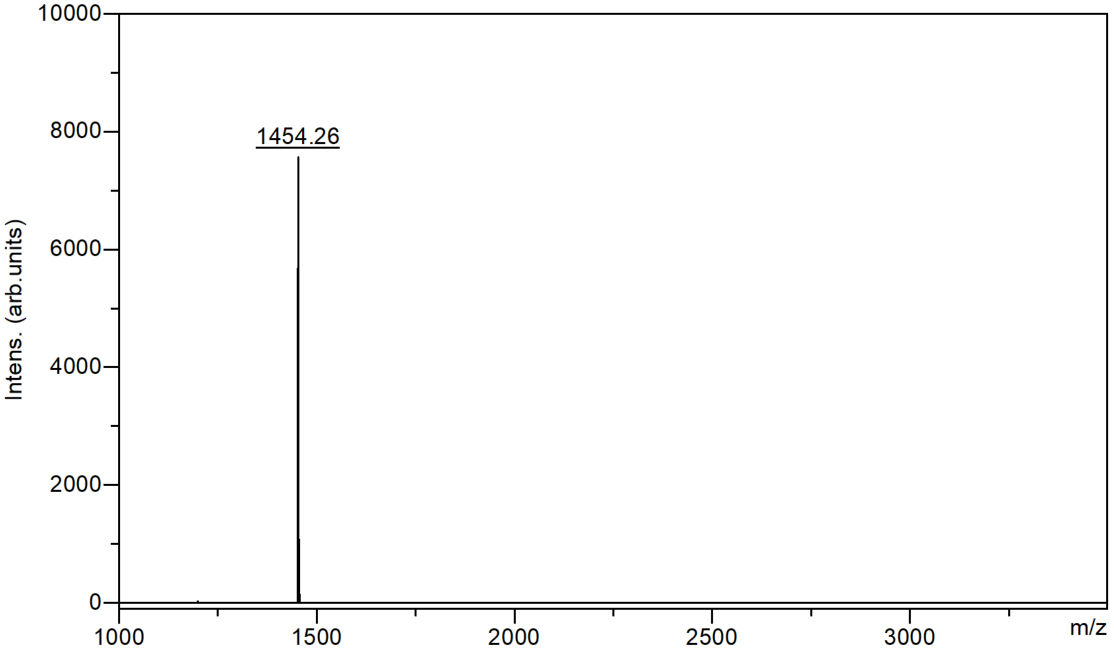

Supplementary Figure 3. MALDI-MS of molecule **2**.

1.2 Synthesis of molecule **1**

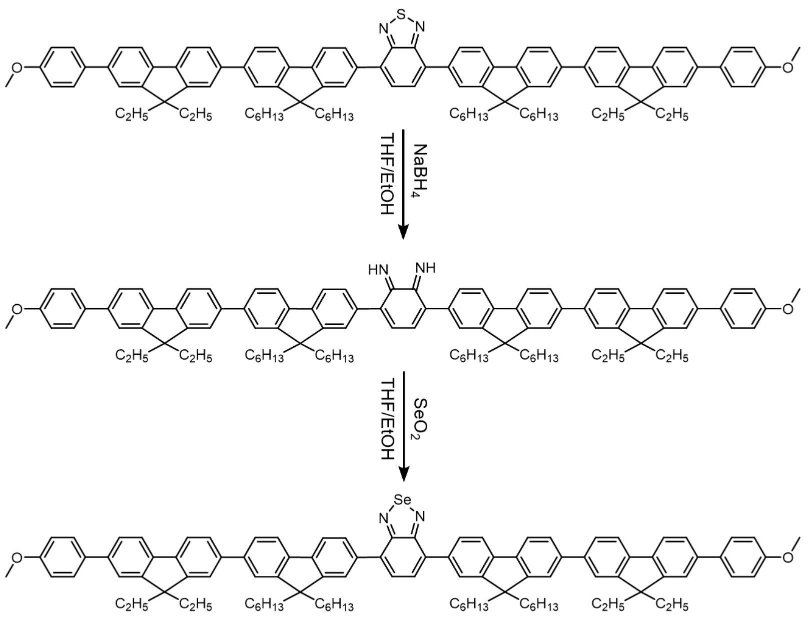

Supplementary Figure. 4 Synthesis route of molecule **1**.

4,7-bis(9',9'-diethyl-9,9-dihexyl-7'-(4-methoxyphenyl)-9H,9'H-[2,2'-bifluoren]-7-yl)benzo[c][1,2,5]selenadiazole (1). To a stirred solution of **2** (110 mg, 0.075 mmol) in a mixture of ethanol (6 mL) and tetrahydrofuran (5 mL), sodium borohydride (57 mg, 1.5 mol) was added at 0 °C. The mixture was then refluxed at 80 °C until the yellow fluorescence of the solution disappeared. After the solvent was evaporated under vacuum, the residue was poured into water (20 mL) and extracted with dichloromethane (3 × 25 mL). The combined organic layer was washed with saturated brine, and dried over Na_2SO_4. Evaporation of the solvent under vacuum gave **3**, which was used directly in the subsequent synthesis without further purification.

To a solution of **3** in a mixture of ethanol (6 mL) and tetrahydrofuran (5 mL), selenium dioxide (167 mg, 1.5 mmol) pre-dissolved in 2 ml of hot water was added. The mixture was stirred for 2 h, followed by evaporation of solvent under vacuum. The residue was poured into water (20 mL) and extracted with dichloromethane (3 × 20 mL). The combined organic layer was washed with saturated brine, dried over Na_2SO_4, and concentrated under vacuum. The residue was purified by column chromatography on silica gel (petroleum: dichloromethane = 1:1 as the eluent) to afford **1** (85 mg, 0.058mmol, 85% yield). The resulting target compound was confirmed by 1H NMR and MALDI-MS as below.

Molecule **1**. ^{1}H NMR (400 MHz, Chloroform-d) δ 8.07 (d, J = 7.8 Hz, 2 H), 7.91 (s, 2 H), 7.84 (m, 10 H), 7.56 (m, 16 H), 7.02 (d, J = 8.2 Hz, 4 H), 3.89 (s, 6 H), 2.17 (d, J = 10.1 Hz, 16 H), 1.17 (d, J = 8.2 Hz, 24 H), 0.87-0.77 (m, 30 H), 0.48 (t, J = 7.3 Hz, 12 H). MALDI-MS: (m/z) = 1501.01.

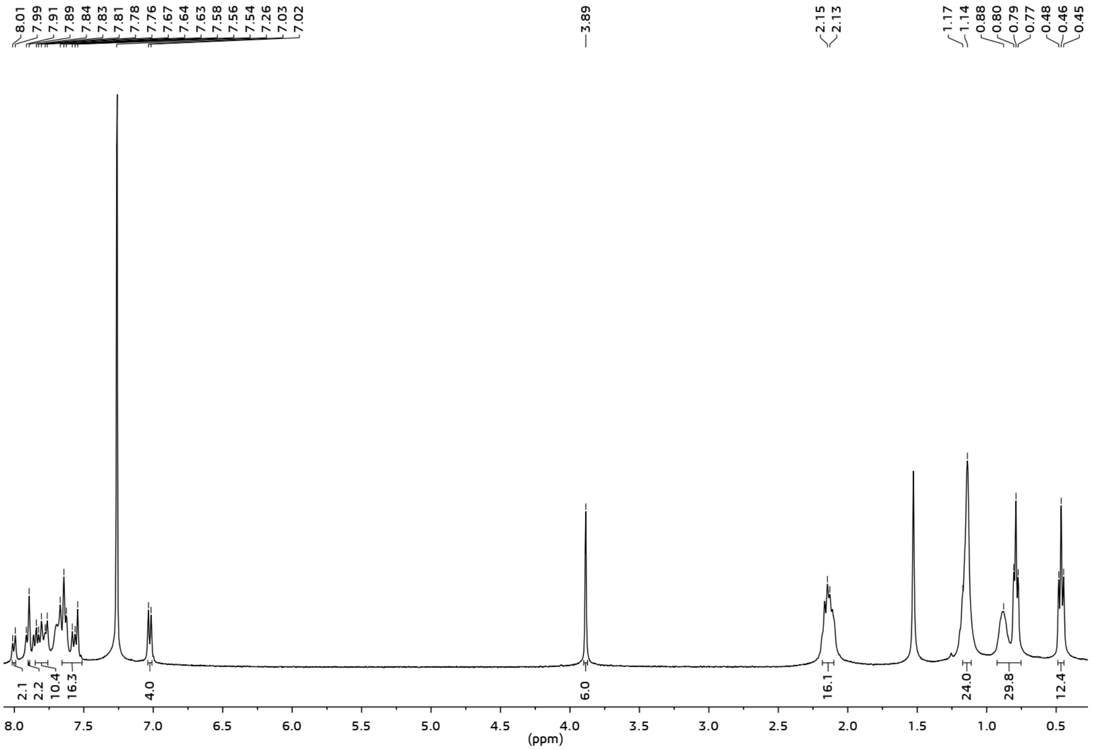

Supplementary Figure 5. ^{1}H NMR of molecule **1**.

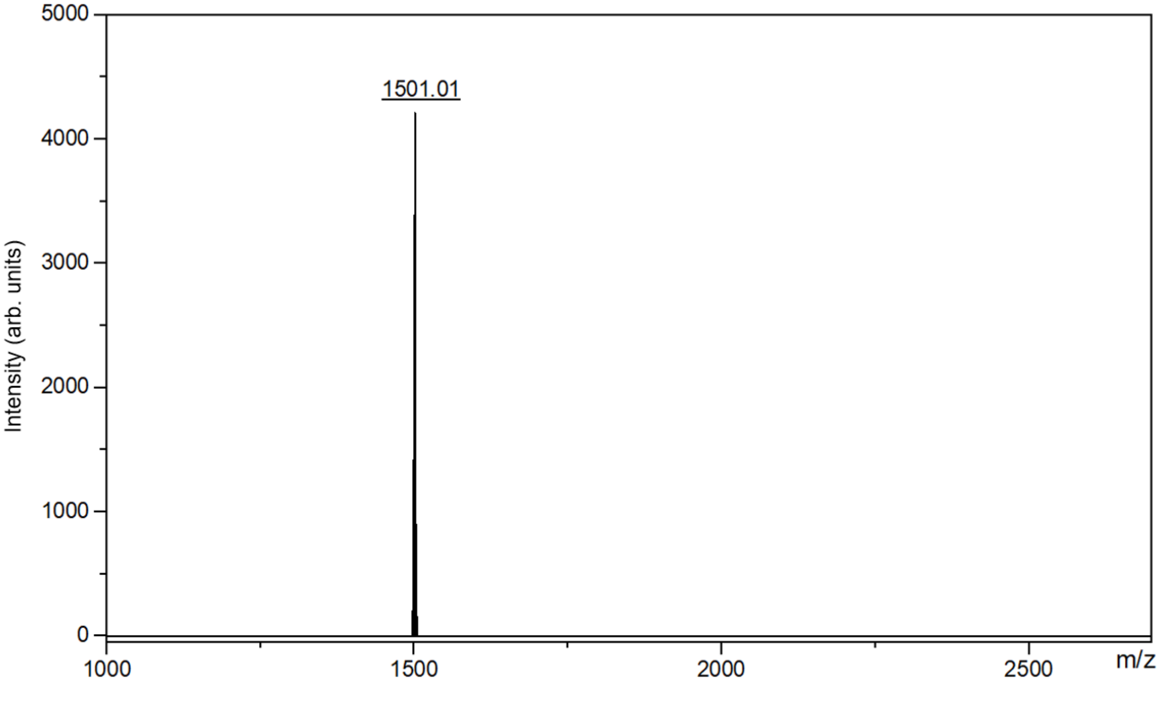

Supplementary Figure 6. MALDI-MS of molecule **1**.

2. Supplementary figures 7 to 24

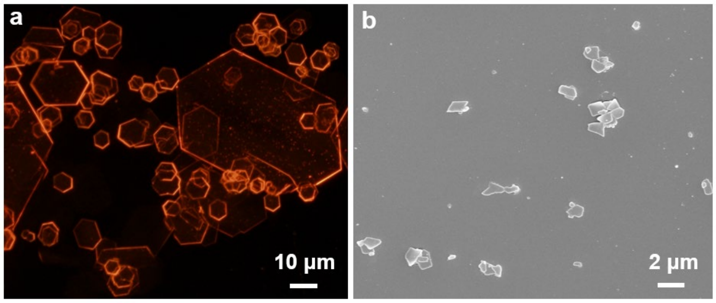

Supplementary Figure 7. (a) Fluorescence-mode optical microscopic images of the original 2D platelets prepared by injecting 1 mL of acetonitrile into a 0.2 mL chloroform solution of **1** (0.5 mg/mL) in a 4 mL vial, followed by aging at 25 °C for 40 h. (b) SEM image of the seeds prepared by sonicating the original platelets at -35 °C for 5 min and then centrifuging at 2000 rpm for 10 min.

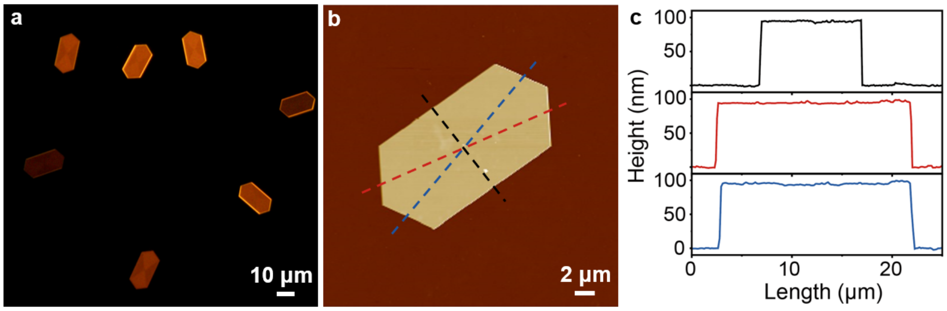

Supplementary Figure 8. (a) Fluorescence-mode optical microscopic images of the thin hexagonal platelets used as the seeds to grow concentric segmented 2D structures. Of note, the discrepancy in self-assembly rates across distinct domains is likely responsible for the packing anisotropy observed in various regions, leading to differing fluorescence intensities between the (100) and (020) platelet domains.[2] (b) AFM height image and (c) the corresponding height profiles of a typical platelet as shown in (a).

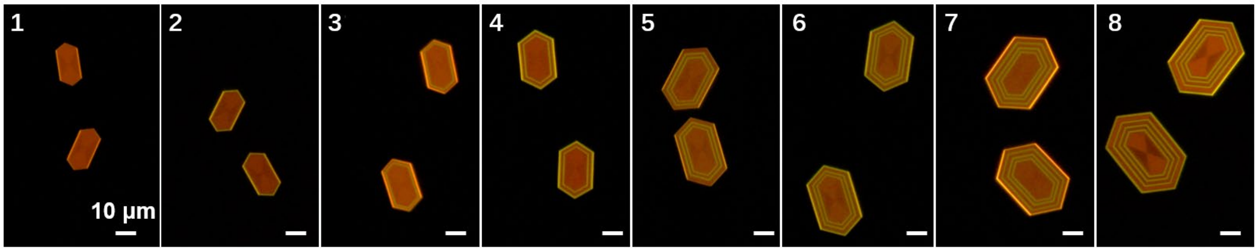

Supplementary Figure 9. Fluorescence-mode optical microscopic images showing the growth of concentric multi-hexagonal 2D platelets by sequential and alternative addition of 100 μL of molecules **1** and **2** (0.024 mg/mL) in a chloroform/acetonitrile mixture (v/v: 1:5).

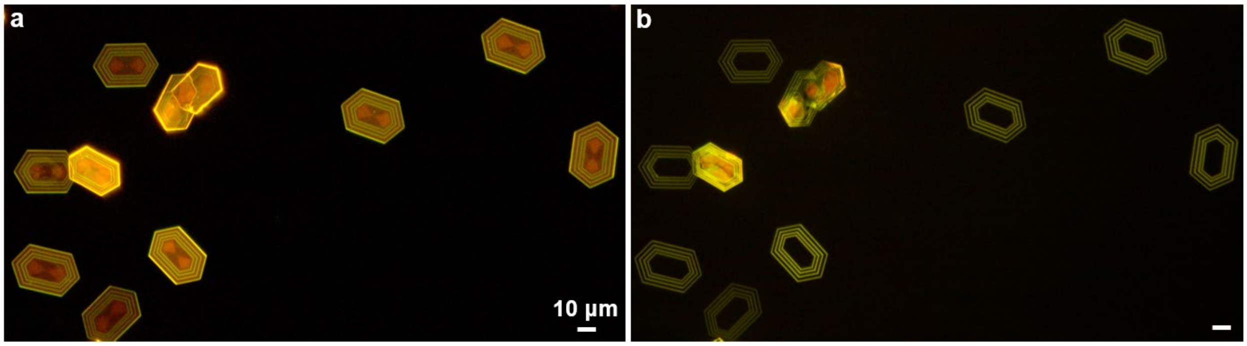

Supplementary Figure 10. Fluorescence-mode optical microscopic images of (a) the concentric multi-hexagonal 2D platelets and (b) the corresponding hollow structures obtained by immersing the precursor platelets in a chloroform/acetonitrile (v/v, 1/5) mixture (2 mL) and exposing to 365 nm UV irradiation (300 mW/cm^2) for 4 min to wash away the segments of **1**.

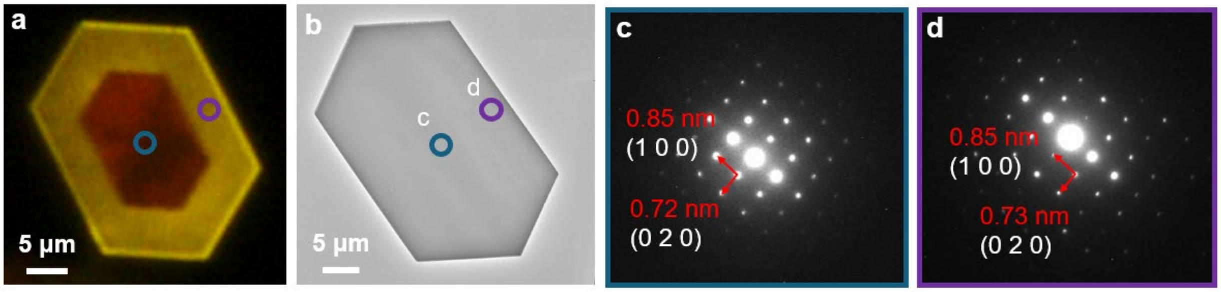

Supplementary Figure 11. (a) Fluorescence-mode optical microscopic image and (b) TEM image of a multi-hexagonal 2D platelet. (c, d) Electron diffraction patterns obtained at two different locations on the 2D platelet, corresponding to segment **1** and **2** as shown in (a) and (b), exhibiting the same d-spacing values along the a and b axes.

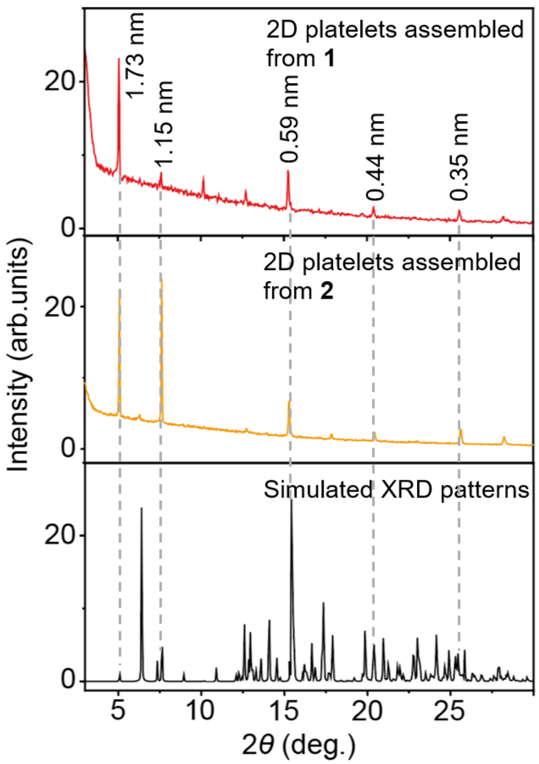

Supplementary Figure 12. Comparing the experimental XRD patterns of the 2D platelets assembled from molecules **1** and **2** with the simulated XRD patterns derived from the single crystal data of molecule **2**.[1]

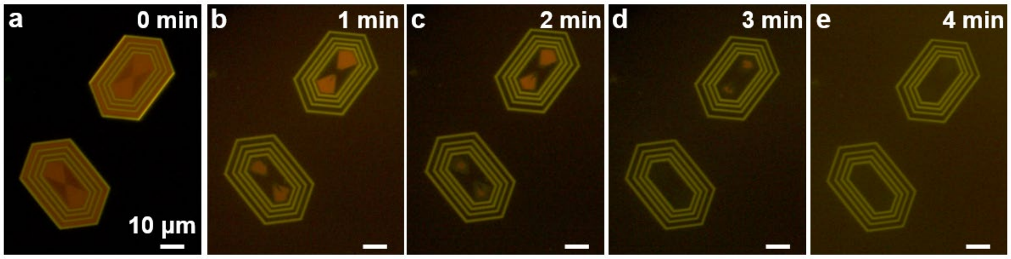

Supplementary Figure 13. (a-e) Fluorescence-mode optical microscopic images showing the gradual removal of segment **1** from the concentric multi-hexagonal 2D platelets immersed in a chloroform/acetonitrile (v/v, 1/5) under UV irradiation (365 nm, 300 mW/cm^2).

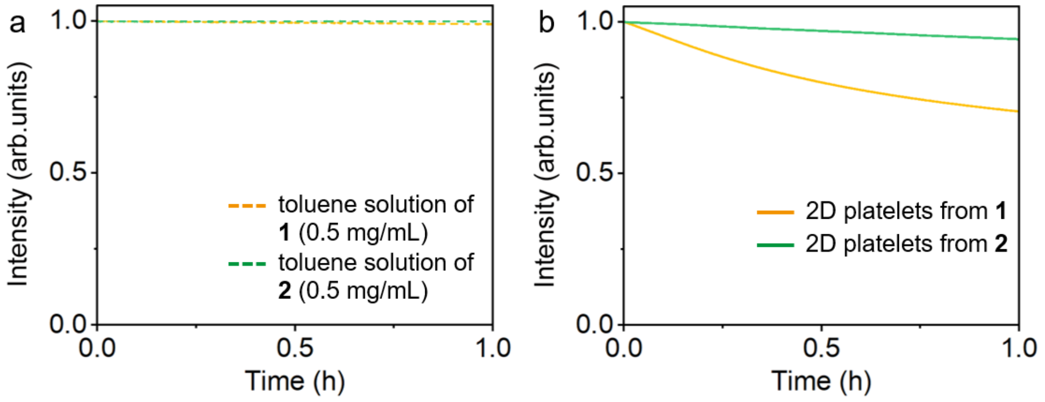

Supplementary Figure 14. (a, b) Fluorescence intensity of molecules **1** and **2** in a toluene solution and their corresponding 2D platelets measured under UV irradiation (385 nm, 3 mW/cm^2). The recorded intensities were based on the emission integration range of 505 to 555 nm for **1** in solution, 570 to 620 nm for 2D platelets from **1**, 535 to 585 nm for **2** in solution, and 550 to 600 nm for 2D platelets from **2**. The distance between the UV light source and the sample was 0.7 cm.

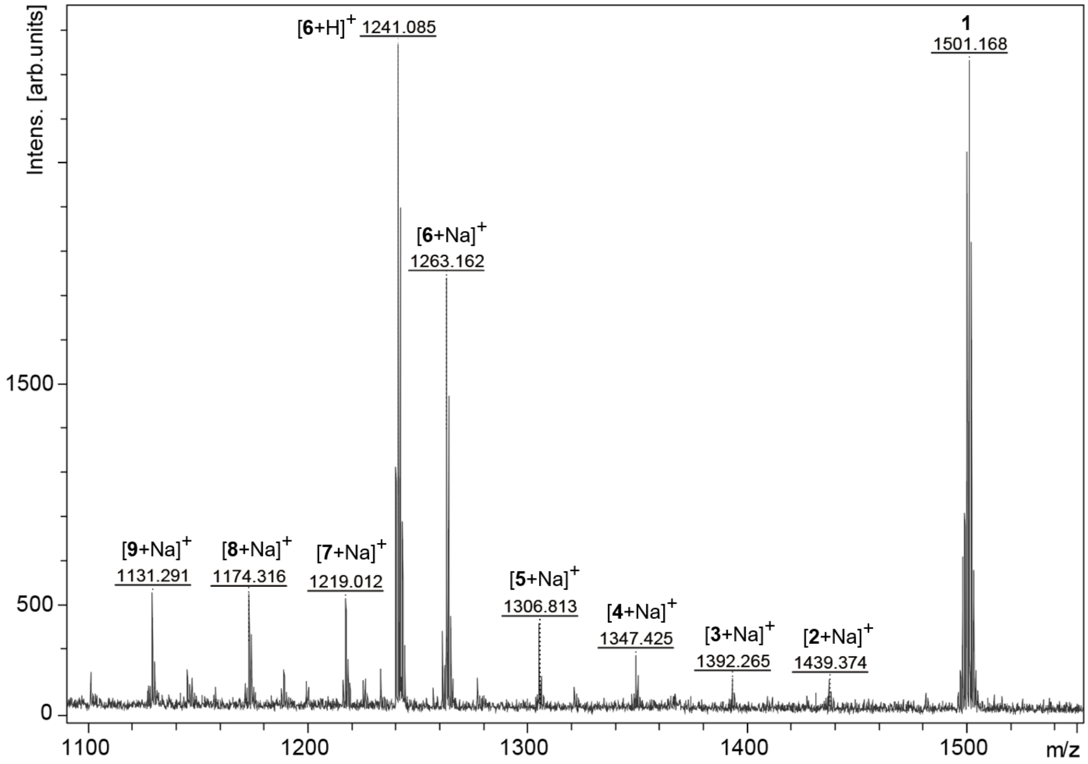

Supplementary Figure 15. MALDI-TOF-MS data obtained from the products resulting from the exposure of 2D platelets assembled from **1** to UV irradiation (365 nm, 300 mW/cm^2) for a duration of 10 min.

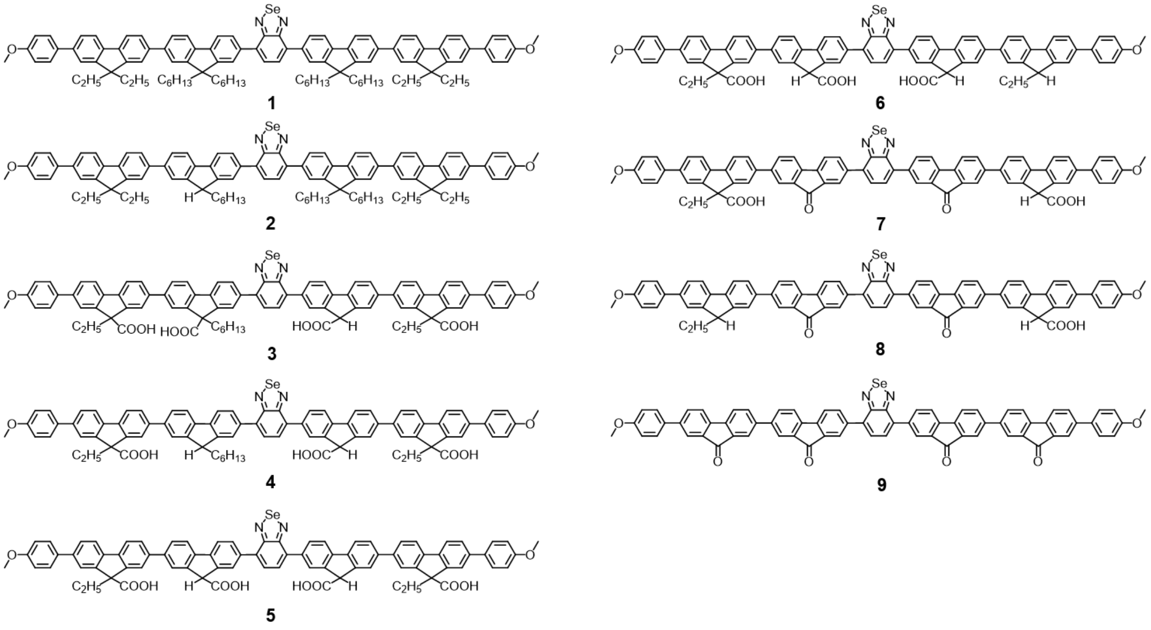

Supplementary Figure 16. A sequence of oxidized products deduced through the MALDI-TOF-MS data above.

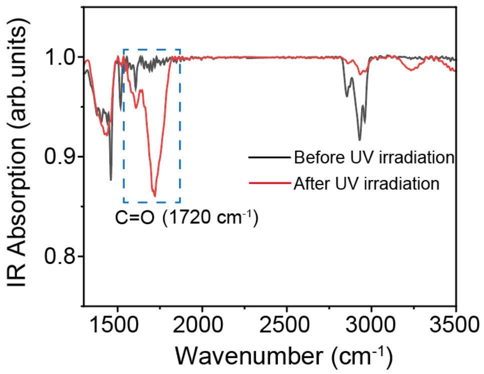

Supplementary Figure 17. IR spectra of the 2D platelets fabricated from molecule **1** before and after 10 min of UV irradiation (365 nm, 300 mW/cm^2).

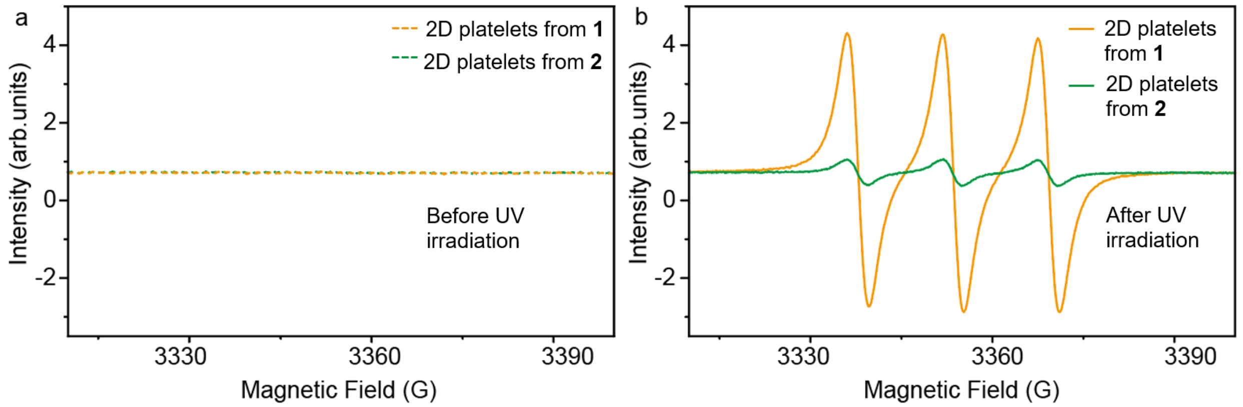

Supplementary Figure 18. EPR spectra of two 2D platelets suspended in an acetonitrile solution (0.5 mg/mL) recorded both before (a) and after (b) 5 min of UV irradiation (365 nm, 300 mW/cm^2) in the presence of triacetonamine hydrochloride (TEMP, 10 mM).

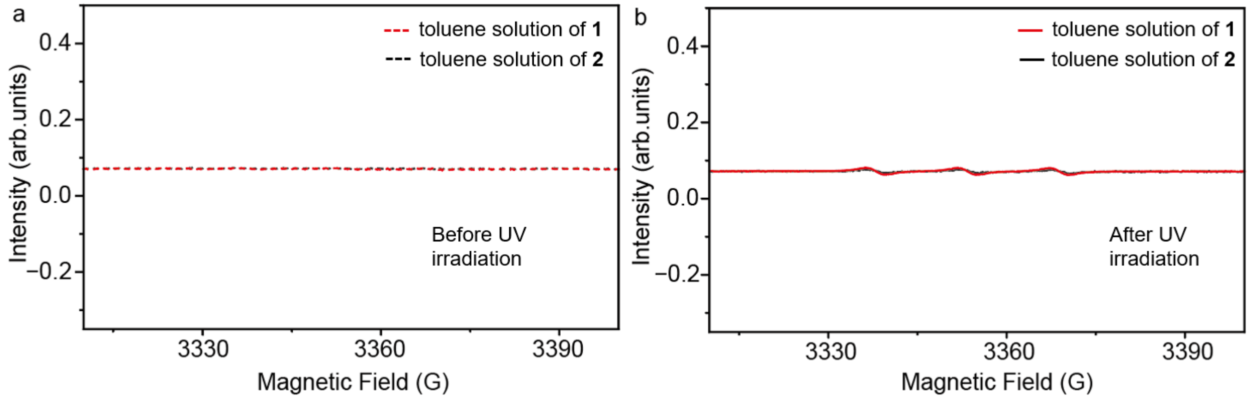

Supplementary Figure 19. EPR spectra of molecules **1** and **2** in a toluene solution (0.5 mg/mL) recorded both before (a) and after (b) 5 min of UV irradiation (365 nm, 300 mW/cm^2) in the presence of TEMP (10 mM).

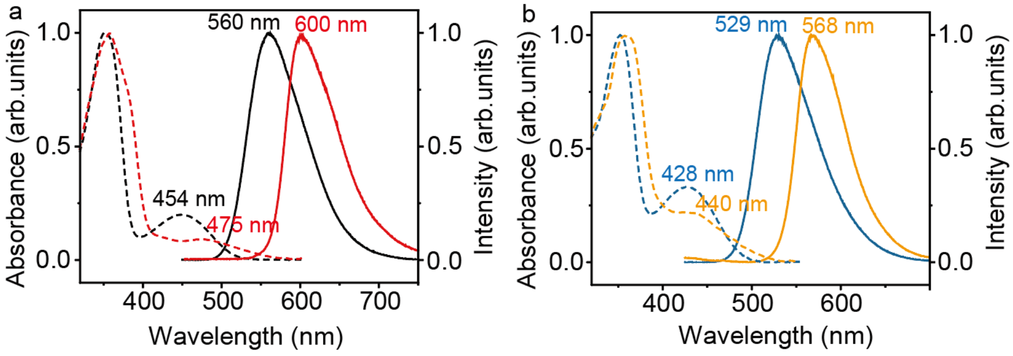

Supplementary Figure 20. (a) Normalized UV-vis absorption spectra (dashed) and fluorescence spectra (solid) of molecule **1** in toluene solution (dark) and 2D platelets from **1** deposited on a glass slide (red). (b) Normalized UV-vis absorption spectra (dashed) and fluorescence spectra (solid) of molecule **2** in toluene solution (blue) and 2D platelets from **2** deposited on a glass slide (orange).

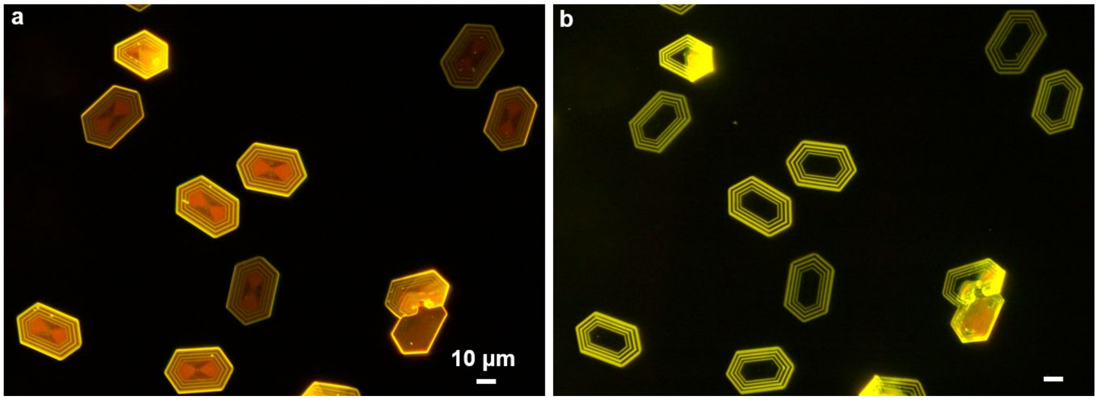

Supplementary Figure 21. Fluorescence-mode optical microscopic images of (a) the concentric multi-hexagonal 2D platelets with segments of **2** of increasing widths and (b) the corresponding hollow structures obtained by immersing the precursor platelets in a chloroform/acetonitrile (v/v, 1/5) mixture (2 mL) and exposing to 365 nm UV irradiation (300 mW/cm^2) for 4 min to wash away the segments of **1**.

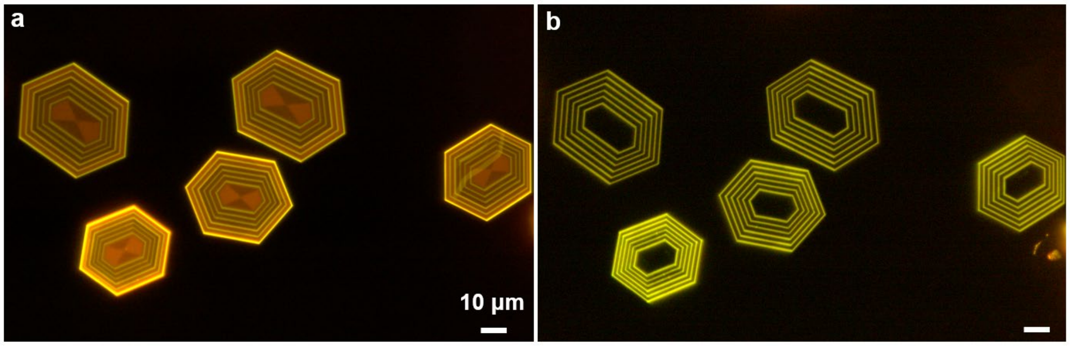

Supplementary Figure 22. Fluorescence-mode optical microscopic images of (a) the concentric multi-hexagonal 2D platelets with six segments of **1** and **2** and (b) the corresponding hollow structures obtained by immersing the precursor platelets in a chloroform/acetonitrile (v/v, 1/5) mixture (2 mL) and exposing to 365 nm UV irradiation (300 mW/cm^2) for 4 min to wash away the segments of **1**.

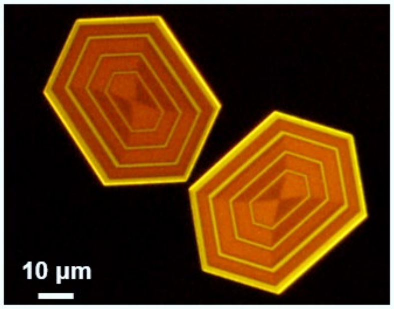

Supplementary Figure 23. Fluorescence-mode optical microscopic image of the concentric multi-hexagonal 2D platelets with segments of **2** of increasing widths corresponding to the hollow 2D platelets in Figure 4b.

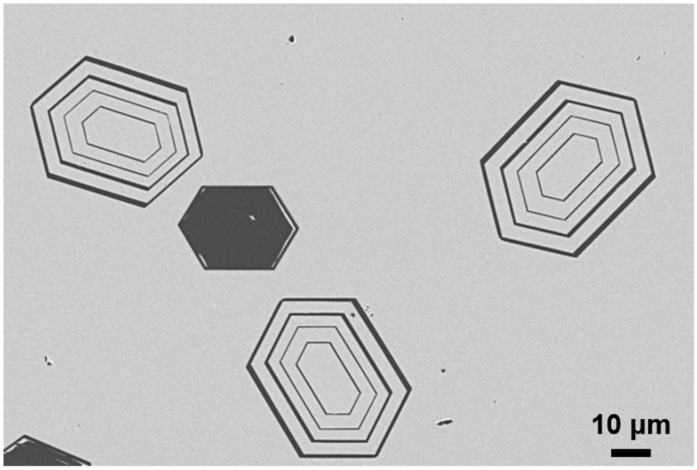

Supplementary Figure 24. SEM images of the concentric multi-hexagonal gold electrodes obtained by washing away the templates through being immersed in chlorobenzene at 120 °C for 24 hours, followed by a rinse with dichloromethane.

3. Additional supplementary figure

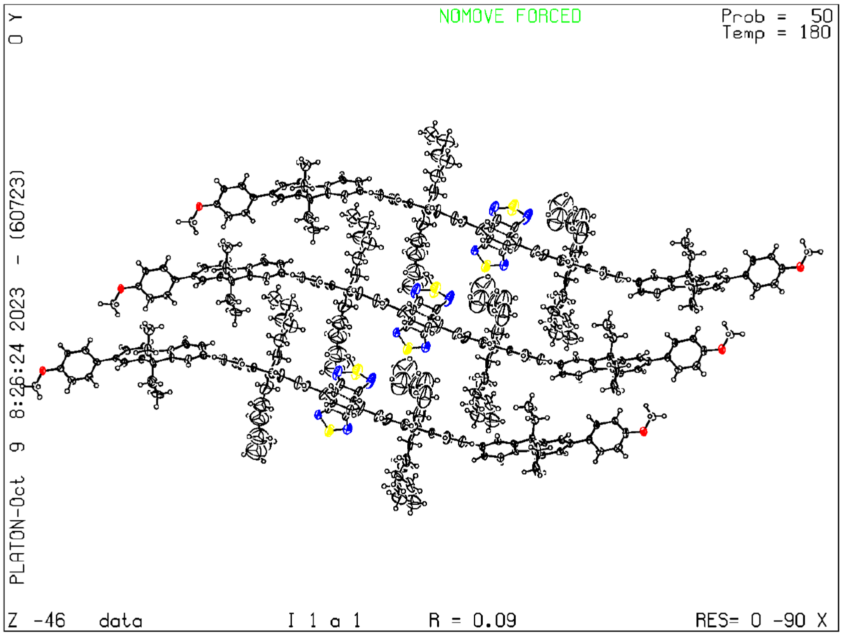

Supplementary Figure 25. The ORTEP-style illustration of single-crystal structures of the 2D platelets assembled from molecules **2**,[1] with Cambridge Crystallographic Data Centre (CCDC) numbers of 2261026.

4. Supplementary References

1. Liao C, *et al.* Living Self-Assembly of Metastable and Stable Two-Dimensional Platelets from a Single Small Molecule. *Chem. Eur. J.*, **29,** e202301747 (2023).

2. H. Y. Jeong, *et al.* Heterogeneous Defect Domains in Single-Crystalline Hexagonal WS_2. *Adv. Mater.* **29,** 1605043 (2017).
